# Wastewater Surveillance for Influenza A Virus and H5 Subtype Concurrent with the Highly Pathogenic Avian Influenza A(H5N1) Virus Outbreak in Cattle and Poultry and Associated Human Cases — United States, May 12–July 13, 2024

**DOI:** 10.15585/mmwr.mm7337a1

**Published:** 2024-09-19

**Authors:** Souci Louis, Miguella Mark-Carew, Matthew Biggerstaff, Jonathan Yoder, Alexandria B. Boehm, Marlene K. Wolfe, Matthew Flood, Susan Peters, Mary Grace Stobierski, Joseph Coyle, Matthew T. Leslie, Mallory Sinner, Dawn Nims, Victoria Salinas, Layla Lustri, Heidi Bojes, Varun Shetty, Elisabeth Burnor, Angela Rabe, Guinevere Ellison-Giles, Alexander T. Yu, Austin Bell, Stephanie Meyer, Ruth Lynfield, Melissa Sutton, Ryan Scholz, Rebecca Falender, Shannon Matzinger, Allison Wheeler, Farah S. Ahmed, John Anderson, Kate Harris, Austin Walkins, Surabhi Bohra, Victoria O’Dell, Virginia T. Guidry, Ariel Christensen, Zack Moore, Erica Wilson, Joshua L. Clayton, Hannah Parsons, Krista Kniss, Alicia Budd, Jeffrey W. Mercante, Heather E. Reese, Michael Welton, Megan Bias, Jenna Webb, Daniel Cornforth, Scott Santibañez, Rieza H. Soelaeman, Manpreet Kaur, Amy E. Kirby, John R. Barnes, Nicole Fehrenbach, Sonja J. Olsen, Margaret A. Honein

**Affiliations:** ^1^Epidemic Intelligence Service, CDC; ^2^Division of Infectious Disease Readiness and Innovation, National Center for Emerging and Zoonotic Infectious Diseases, CDC; ^3^Influenza Division, National Center for Immunization and Respiratory Diseases, CDC; ^4^Department of Civil & Environmental Engineering, School of Engineering and Doerr School of Sustainability, Stanford University, Stanford, California; ^5^Gangarosa Department of Environmental Health, Rollins School of Public Health, Emory University, Atlanta, Georgia; ^6^Michigan Department of Health and Human Services; ^7^Illinois Department of Public Health; ^8^Texas Department of State Health Services; ^9^Center for Infectious Diseases, Division of Communicable Disease Control, California Department of Public Health, Richmond, California; ^10^Minnesota Department of Health; ^11^Public Health Division, Oregon Health Authority, Portland, Oregon; ^12^Oregon Department of Agriculture; ^13^Oregon State University; Corvallis, Oregon; ^14^Colorado Department of Public Health & Environment; ^15^Kansas Department of Health and Environment; ^16^City of Boise, Boise, Idaho; ^17^Central District Health; ^18^North Carolina Department of Health and Human Services; ^19^South Dakota Department of Health; ^20^Global Government Solutions Corporation, San Antonio, Texas; ^21^Chenega Corporation, Atlanta, Georgia.

**Summary**

**What is already known about this topic?**

Wastewater surveillance can detect influenza A virus and the H5 subtype, although current testing does not distinguish between human and animal sources.

**What is added by this report?**

During May 12–July 13, 2024, high influenza A virus levels were detected in wastewater in four states, including three states with seasonal human influenza virus activity noted during this time. The H5 subtype was detected in wastewater in nine states; follow-up investigations in many of these states revealed likely animal-related sources, including those related to milk processing.

**What are the implications for public health practice?**

Early work to interpret influenza A virus and H5 subtype detections in wastewater can help with public health preparedness and response for the upcoming respiratory illness season.

## Abstract

As part of the response to the highly pathogenic avian influenza A(H5N1) virus outbreak in U.S. cattle and poultry and the associated human cases, CDC and partners are monitoring influenza A virus levels and detection of the H5 subtype in wastewater. Among 48 states and the District of Columbia that performed influenza A testing of wastewater during May 12–July 13, 2024, a weekly average of 309 sites in 38 states had sufficient data for analysis, and 11 sites in four states reported high levels of influenza A virus. H5 subtype testing was conducted at 203 sites in 41 states, with H5 detections at 24 sites in nine states. For each detection or high level, CDC and state and local health departments evaluated data from other influenza surveillance systems and partnered with wastewater utilities and agriculture departments to investigate potential sources. Among the four states with high influenza A virus levels detected in wastewater, three states had corresponding evidence of human influenza activity from other influenza surveillance systems. Among the 24 sites with H5 detections, 15 identified animal sources within the sewershed or adjacent county, including eight milk-processing inputs. Data from these early investigations can help health officials optimize the use of wastewater surveillance during the upcoming respiratory illness season.

## Introduction

Wastewater surveillance is used to monitor human shedding of pathogens, including SARS-CoV-2, at a community level and is independent of symptoms, testing access, and care-seeking behavior (*1*). Some sites have conducted wastewater influenza virus surveillance for several years, and findings have correlated with traditional influenza surveillance measures (*2*–*6*). The zoonotic outbreak of highly pathogenic avian influenza (HPAI) A(H5N1) in the United States has resulted in 13 confirmed human cases during January–August 2024.* As part of the response to this outbreak, CDC and state and local health departments are using wastewater surveillance to monitor influenza A virus and the H5 subtype; however, current testing techniques cannot distinguish between human and animal sources. This report summarizes data from the first 9 weeks of monitoring influenza A virus and the H5 subtype in wastewater across the United States, including findings from collaborations with state and local health departments to investigate potential sources during the ongoing H5N1 public health response.

## Methods

### Influenza A Virus Testing

Wastewater samples collected from approximately 750 sites in 48 states and the District of Columbia during May 12–July 13, 2024, were tested for influenza A virus by state and local health departments, a CDC contractor, or an academic partner program (WastewaterSCAN [https://www.wastewaterscan.org/en]); and results were submitted to CDC’s Data Collation and Integration for Public Health Event Response (DECIPHER) pipeline.^†^ Although not specific to the H5N1 subtype, the influenza A virus testing performed routinely by partners across the United States frequently detects any influenza A virus, including seasonal and H5 subtypes. For this analysis, concentrations of influenza A virus in wastewater were measured using digital polymerase chain reaction testing with various primer and probe oligonucleotides and assay conditions that were optimized by each laboratory (*7*). For each site, the percentile of the most recent week’s normalized concentration was calculated compared with the normalized concentrations reported during October 1, 2023–March 2, 2024, corresponding to the portion of the influenza season before the reported HPAI A(H5N1) outbreak in dairy cattle. Influenza A virus levels at each site were categorized as high, above average, moderate, low, or minimal.^§^

### Influenza A(H5) Virus Subtype Testing

In April 2024, a digital polymerase chain reaction assay for the H5 hemagglutinin gene of the influenza A virus was developed and evaluated by WastewaterSCAN for testing wastewater and detected H5 viral RNA in wastewater samples from multiple locations experiencing cattle outbreaks (*7*). In May 2024, routine H5 testing was implemented at 193 sites (reduced to 152 sites by July 1, 2024) across 41 states, and results were displayed on a public dashboard^¶^; H5 testing was also implemented at 10 additional sites in one state, for a total of 203 sites with any H5 testing. CDC’s wastewater DECIPHER data pipeline was updated to receive influenza A virus subtyping results, and submission of H5 virus data commenced in July 2024.

### Collaboration To Evaluate Wastewater Signals

CDC notified jurisdictions of high influenza A virus levels on a weekly basis; notification of new H5 detections were provided daily. CDC provided jurisdictions with a checklist** for follow-up, which included reviewing human influenza surveillance systems and characterizing sewershed inputs (substances that flow into sewer pipes) in partnership with wastewater utilities, departments of agriculture, and others. This activity was reviewed by CDC, deemed not research, and was conducted consistent with applicable federal law and CDC policy.^††^

## Results

### Detection of High Influenza A Virus Levels

Among an average of 309 wastewater sites with sufficient data for analysis for ≥1 week during May 12–July 13 (Figure), 11 sites in four states (California, Illinois, Kansas, and Oregon) had high influenza A levels detected at least once (Table). In three of these states, six wastewater sites with high influenza A virus levels were in communities with evidence of human influenza activity, based on other surveillance. The influenza A virus wastewater data are not specific to H5, and none of these four states reported H5 human influenza cases, nor did they report any confirmed cases in livestock herds or poultry within their sewersheds or counties during this time.^§§^ Most of these sites reported open or combined sewersheds, and some sites identified specific potential sources of animal input.^¶¶^

**FIGURE:**
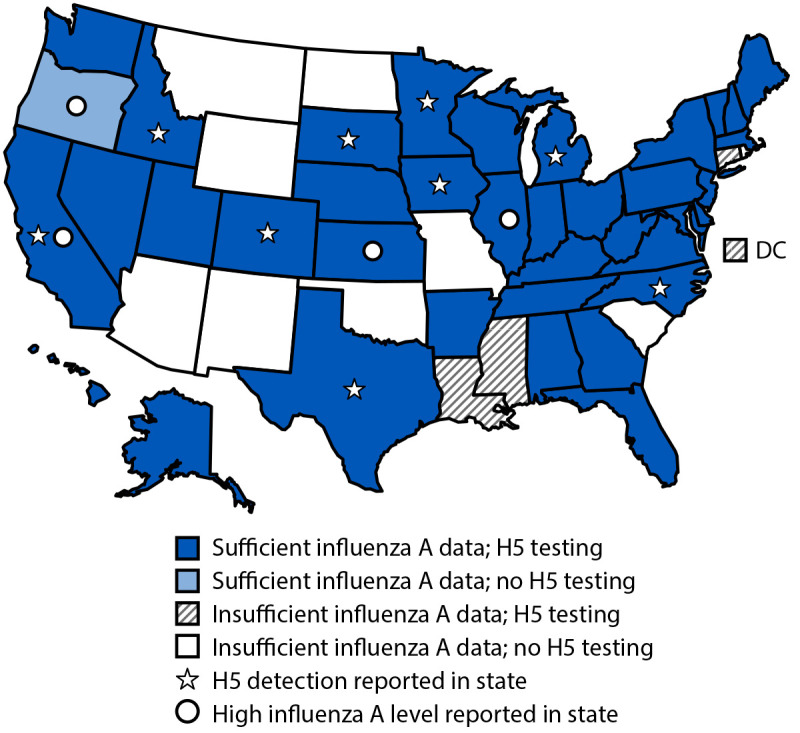
Influenza A virus and H5 subtype testing in wastewater and sites with high levels* of influenza A virus or H5 detections reported to CDC — United States, May 12–July 13, 2024^†^ Abbreviation: DC = District of Columbia. * Influenza A levels were categorized as being at a high (≥80th percentile compared with previous influenza season), above average (60th to <80th percentile), moderate (40th to <60th percentile), low (20th to <40th percentile), or minimal (<20th percentile) level, or as having insufficient data for analysis. https://www.cdc.gov/nwss/about-data.html ^†^ Sites with sufficient data for analysis included those that had influenza A wastewater testing data before October 1, 2023, had 10 or more samples in which influenza A was detected during October 1, 2023–March 2, 2024, had six or more samples tested during October 1, 2023–January 1, 2024, and submitted data in the 2 weeks before analysis.

**TABLE:** Characteristics of sites with high* influenza A virus activity or H5 detection in wastewater — United States, May 12–July 13, 2024

Characteristic	No. (%)			
	Influenza A virus testing^†^		H5 subtype testing^§^	
	States with high level^¶^ n = 4	Sites with high level n = 11	States with H5 detections** n = 9	Sites with H5 detections n = 24
**Human influenza surveillance signals**				
Evidence of human influenza activity based on other surveillance systems	3 (75)	6 (54)	1 (11)	3 (13)
**Type of sewershed**				
Combined (open)	NA	8 (73)	NA	5 (21)
Separate (closed)	NA	3 (27)	NA	17 (71)
Information not provided	NA	0 (—)	NA	2 (8)
**Potential signal source (within sewershed or county)^††^**				
**Dairy or livestock**				
Dairy operations	0 (—)	0 (—)	3 (33)	5 (21)
Livestock truck wash	2 (50)	2 (18)	1 (11)	3 (13)
Milk processing	3 (75)	3 (27)	4 (44)	8 (33)
Meat processing	0 (—)	0 (—)	5 (56)	7 (29)
No dairy or livestock inputs identified	4 (100)	5 (45)	4 (44)	10 (42)
Information not provided	1 (9)	1 (9)	1 (11)	2 (8)
**Avian**				
Wild bird inputs suspected as possible	2 (50)	6 (55)	4 (44)	10 (42)
Active poultry cases	0 (—)	0 (—)	2 (22)	2 (8)
No avian inputs identified	2 (50)	4 (36)	6 (67)	11 (46)
Information not provided	1 (9)	1 (9)	1 (11)	2 (8)
Any dairy, livestock, or avian input	3 (75)	8 (73)	8 (89)	15 (63)
Information not provided	1 (9)	1 (9)	1 (11)	2 (8)
**Other**				
Identified human influenza H5 cases during this period	0 (—)	NA	2 (22)^§§^	NA
H5 confirmed cases in livestock herds during this period	0 (—)	NA	7 (78)^¶¶^	NA

**Abbreviation:** NA = not applicable.

* Influenza A levels were categorized as being at a high (≥80th percentile compared with previous influenza season), above average (60th to <80th percentile), moderate (40th to <60th percentile), low (20th to <40th percentile), or minimal (<20th percentile) level, or as having insufficient data for analysis. https://www.cdc.gov/nwss/about-data.html

^†^ At an average of 309 weekly sites in 38 states with sufficient data for analysis.

^§^ At 203 sites in 41 states.

^¶^ The following jurisdictions had one or more sites with a high influenza A level during the 9-week period: California, Illinois, Kansas, and Oregon.

** The following jurisdictions had one or more sites with an H5 detection during the 9-week period: California, Colorado, Idaho, Iowa, Michigan, Minnesota, North Carolina, South Dakota, and Texas.

^††^ Each site had the option to select multiple potential sources; thus, these categories and responses within categories are not mutually exclusive.

^§§^ Colorado and Michigan had identified human cases of influenza A(H5N1) virus infection during this period.

^¶¶^ Colorado, Idaho, Iowa, Michigan, Minnesota, South Dakota, and Texas had publicly reported influenza A(H5N1) virus infections in dairy cattle herds during this period.

### H5 Subtype Detections

Among 41 states with influenza A H5 testing in wastewater, nine states (California, Colorado, Idaho, Iowa, Michigan, Minnesota, North Carolina, South Dakota, and Texas) reported one or more H5 detections during May 12–July 13, and 32 states did not have H5 detections during this period. The nine states with H5 detections in wastewater included seven states with an HPAI A(H5N1)–infected herd reported during this period and one additional state with an infected herd reported before this period. Two of these nine states (Colorado and Michigan) reported confirmed human cases of HPAI A(H5N1) virus infection during this time.*** Among the 32 states with H5 testing in wastewater and no H5 detections in wastewater during the analysis period, 30 (94%) had no herds with HPAI A(H5N1) virus infections reported during this time, and two had infected herds reported only before this period.

In determining inputs to specific wastewater sites, 15 of 24 sites (63%) with H5 detections identified some animal inputs within the sewershed or county. Eight of 24 sites (33%) identified milk-processing inputs within the sewershed catchment area. Additional inputs noted were meat processing, dairy operations within the sewershed or adjacent county, other sources of livestock waste (e.g., truck wash), wild bird inputs, and domestic poultry farms within the sewershed or county.^†††^

## Discussion

During May 12–July 13, most U.S. wastewater sites tested did not have high influenza A virus levels or any detections of the H5 subtype. Among the sites that did have high influenza A virus levels, the most frequent finding was corresponding evidence of human influenza A virus activity in the community. Among sites that reported H5 detections in wastewater, all but one were in a state with reported infected dairy herds during or before the surveillance period, and animal-related inputs such as effluent from milk-processing plants and suspected contributions from wild birds were frequently reported. These differences highlight the importance of influenza A virus subtyping to provide data specific to HPAI outbreaks.

The findings in this report underscore the importance of using a One Health approach that leverages multisectoral collaboration to understand the complexity of inputs from animal and human sources.^§§§^ The current zoonotic outbreak of HPAI A(H5N1) virus highlights the importance of coordination across health, agriculture, wildlife, food safety, and other partners. Investigations into wastewater signals also require coordination among public health, academic, municipal water treatment, and community partners. Influenza A(H5N1) virus has been found at very high concentrations in milk from infected cows (*8*); investigations of influenza A and H5 virus signals in wastewater suggest that milk effluent from milk-processing facilities can be a major contributor to H5 viral particles in wastewater (*7*,*9*). Monitoring influenza A viruses and specific influenza A virus subtypes in wastewater might serve as One Health surveillance indicators of the presence of influenza viruses in a community. Further, existing human clinical influenza surveillance systems are essential for quickly identifying H5 virus infections in humans and monitoring seasonal influenza virus activity.

Public health agencies that conduct wastewater monitoring to complement influenza surveillance systems should be prepared to add influenza A virus subtype testing when needed to improve understanding of influenza A virus detections in the context of the current HPAI A(H5N1) outbreak. CDC-funded National Wastewater Surveillance System’s Centers of Excellence are expanding influenza A virus testing and subtyping, which can contribute to source investigations and be deployed at strategic times and places.^¶¶¶^ Although this report focused on comparing influenza A virus levels with those from the previous season and identifying H5 detections, subtyping for seasonal influenza A(H1) and A(H3) viruses in wastewater samples and using state and national viral activity levels to monitor influenza A across sites can help with interpretation of wastewater surveillance data during the upcoming influenza season.****

A critical need for clear communication about the meaning of detection of influenza A virus and subtypes exists. Data dashboards can provide regular wastewater surveillance updates to the public, the media, and health care providers; however, these data need to be accompanied by clear public health interpretations focusing on potential human risk and public health actions, which could include alerts to health care providers or increasing availability of testing or vaccines as has been done for SARS-CoV-2 and mpox. Activities to monitor influenza A virus and subtypes using wastewater data are likely to evolve as the methodologies and interpretation are further evaluated and refined.

### Limitations

The findings in this report are subject to at least five limitations. First, although influenza viruses can be detected in wastewater, current techniques cannot distinguish between human and animal sources, and the current approach for H5 testing in wastewater is not specific to HPAI A(H5N1) viruses; H5 detections in wastewater might reflect animal rather than human infections and might be detection of low pathogenic avian influenza rather than HPAI A(H5N1) viruses. Second, limited data are available regarding the proportion of persons infected with influenza viruses who shed virus in urine or feces, and how the concentration of viral shedding varies by subtype and across the course of illness (*10*). Third, population wastewater surveillance coverage varies substantially by state; therefore, data are most informative when used in conjunction with clinical human influenza surveillance data. Fourth, states reported information on the investigations of sewershed inputs for sites with high influenza A virus levels or H5 detections, but comparison information for sites without these signals was not collected; therefore, epidemiologic measures for these possible associations could not be generated. Finally, the comprehensiveness of data collection after a signal in wastewater varied widely. Public health investigations into potential sources of H5 viruses in wastewater can be complex (e.g., milk-processing inputs can include milk from other states) and might support or refute likely sources of H5 without providing definitive conclusions.

### Implications for Public Health Practice

Lessons learned during early follow-up investigations of wastewater signals can help health officials implement an improved measure of influenza A virus levels in wastewater and optimize the use of wastewater surveillance during the upcoming respiratory illness season. The findings in this report, and data from wastewater surveillance in general, can complement traditional influenza surveillance systems. A One Health approach with multisectoral collaboration and data-informed guidance on when and how to use influenza virus subtyping of wastewater might enhance the public health response to the current outbreak.

## Footnotes

*
https://www.cdc.gov/bird-flu/situation-summary/index.html

†
https://archive.cdc.gov/#/details?url=https://www.cdc.gov/nwss/reporting.html

§ Influenza A levels were categorized as being at a high (≥80th percentile compared with previous influenza season), above average (60th to <80th percentile), moderate (40th to <60th percentile), low (20th to <40th percentile), or minimal (<20th percentile) level, or as having insufficient data for analysis. https://www.cdc.gov/nwss/about-data.html

¶
https://data.wastewaterscan.org/

** Checklist is available at https://stacks.cdc.gov/view/cdc/160378.

†† 45 C.F.R. part 46, 21 C.F.R. part 56; 42 U.S.C. Sect. 241(d); 5 U.S.C. Sect. 552a; 44 U.S.C. Sect. 3501 et seq.

§§ https://www.cdc.gov/bird-flu/spotlights/h5n1-response-07262024.html; https://www.aphis.usda.gov/livestock-poultry-disease/avian/avian-influenza/hpai-detections/hpai-confirmed-cases-livestock

¶¶ An open system (combined sewer) includes both sewage and stormwater runoff; a closed system (separate sanitary sewer) should only include sewage.

*** Colorado, Idaho, Iowa, Michigan, Minnesota, South Dakota, and Texas had publicly reported influenza A(H5N1) virus infections in dairy cattle herds during this time. Colorado and Michigan had publicly reported human cases during this period.

††† Migratory birds were a possible contributor to inputs at 10 (42%) of 24 sites.

§§§
https://www.cdc.gov/one-health/about/index.html

¶¶¶ More information about the CDC-funded National Wastewater Surveillance System’s Centers of Excellence can be found online. https://www.cdc.gov/nwss/centersofexcellence.html

**** More information about the SARS-CoV-2 and influenza A Wastewater Viral Activity Levels can be found online. https://www.cdc.gov/nwss/about-data.html#data-method
